# Self-esteem and suppression of negative emotions among medical students: a cross-sectional study

**DOI:** 10.3389/fpubh.2026.1846894

**Published:** 2026-08-04

**Authors:** Anna Knyszyńska, Marcin Grajek, Paulina Zabielska, Marek Landowski, Krzysztof Zdziarski, Beata Karakiewicz

**Affiliations:** 1 Department of Humanities and Occupational Therapy, Pomeranian Medical University in Szczecin, Szczecin, Poland; 2 Subdepartment of Social Medicine and Public Health, Department of Social Medicine, Pomeranian Medical University in Szczecin, Szczecin, Poland; 3 Independent Research and Biostatistics Laboratory, Department of Social Medicine, Pomeranian Medical University in Szczecin, Szczecin, Poland

**Keywords:** personality psychology, self-esteem, suppression of emotions, survey research, young adults

## Abstract

**Background/objectives:**

Suppression of negative emotions is one of the response-focused strategies within the broader field of emotion regulation. It refers to the inhibition or restriction of emotional expression, particularly in relation to anger, anxiety, and depressed mood. Among medical students, this issue may be especially relevant because professional socialization often promotes emotional restraint and functioning under psychological pressure. The aim of this study was to assess the association between self-esteem and suppression of negative emotions among medical students in Poland. Selected self-reported psychosocial characteristics were also analyzed as contextual variables.

**Methods:**

A cross-sectional study was conducted among 611 students of medical and health sciences universities in Poland. Data were collected online using the Computer Assisted Web Interview procedure. The Rosenberg Self-Esteem Scale (SES), the Courtauld Emotional Control Scale (CECS), and an original sociodemographic questionnaire were used.

**Results:**

The mean SES score was 28.22 ± 5.42, indicating an average level of self-esteem, while the mean CECS score was 53.59 ± 12.49. Self-esteem was negatively correlated with the total CECS score and with all CECS subscales: anger, depression, and anxiety. Differences in self-esteem and suppression of negative emotions were also observed according to selected self-reported psychosocial characteristics. A sense of belonging to a social group was associated with higher self-esteem and lower CECS scores. Secondary exploratory indirect-association analyses showed that CECS anger, depression, and anxiety subscales were strongly interrelated. These findings were interpreted as hypothesis-generating and not as evidence of causal mediation or temporal mechanisms.

**Conclusion:**

Lower self-esteem was associated with greater suppression of negative emotions among medical students. Selected self-reported psychosocial characteristics were associated with differences in SES and CECS scores, but these findings should be interpreted descriptively and with caution. Due to the cross-sectional design, the findings should be interpreted as associations and not as evidence of causal relationships.

## 1 Introduction

In response to environmental factors, people manifest various reactions that are regulated by biological and behavioral processes. Biological reactions are related to the systemic, including hormonal, response of the organism to the external factor. Behavioral reactions are related to the previous experiences of the individual (1).

The psychosocial condition of modern man is based on the need to constantly adapt to the dynamically changing conditions of the external environment. Emotions are neurobiological states that are the result of the sum of an individual’s perception of reality and the reaction of his or her body to the action of an external factor. In the process of maturation, a person learns to regulate the emotions he feels, show them appropriately, and express them in various ways. After the end of biological maturation, the human body is prepared to physically react to external stimuli and respond to them through hormonal or physical-behavioural reactions, which are related, among other things, to the showing and expression of emotions (2).

Regulation and control of emotions refers to all situations in which a person influences his or her emotions and moderates their expression depending on the need (3–6). Emotion regulation is a broad, multidimensional construct that includes different strategies used to influence the occurrence, intensity, duration, and expression of emotions. Within this broader framework, suppression is usually classified as a response-focused strategy, because it involves inhibiting or restricting emotional expression after an emotional response has already been activated (7). Therefore, suppression should not be treated as synonymous with emotion regulation as a whole. In the present study, the term “suppression of negative emotions” is used to refer specifically to the construct measured by the Courtauld Emotional Control Scale, namely the control or inhibition of the expression of anger, anxiety, and depressed mood. How emotions are regulated may be influenced by the individual’s level of self-acceptance, presented worldview models and subjective self-esteem (4). The indicated factors are interesting research areas from a global perspective on the human psychosocial condition.

Conscious regulation of emotions and self-esteem may be related to psychosocial functioning, but the meaning of emotional control depends on the specific regulatory strategy being considered. In particular, suppression of emotional expression should not be automatically interpreted as adaptive regulation. Although suppression may reduce visible emotional expression, it may also be associated with psychological, social, and physiological costs (8). Self-esteem may be understood as an important psychological resource that shapes the way individuals interpret themselves, their social experiences, and their emotional reactions. Lower self-esteem may be associated with greater sensitivity to interpersonal threat, discrimination, and social exclusion, which in turn may be related to a stronger tendency to inhibit the expression of negative emotions. This association may be particularly relevant among medical students, who are exposed to academic pressure, contact with suffering, professional expectations, and norms of emotional restraint. Previous studies suggest that low self-esteem in young people exposed to stigma, prejudice, or discrimination may be an important target for psychological support (9). Self-esteem may be considered an important psychosocial resource associated with emotional functioning and social adaptation.

Analyzing self-esteem and suppression of negative emotions may help to better understand selected aspects of psychosocial functioning among medical students. Although previous studies have examined self-esteem, trauma exposure, social exclusion, and emotion regulation separately, less attention has been paid to their combined association with suppression of negative emotions among medical students. This is important because medical students are exposed to academic stress, professional expectations, contact with illness and suffering, and social norms that may promote emotional restraint (10, 11). Therefore, examining how self-esteem, adverse psychosocial experiences, and social belonging are associated with suppression of negative emotions may contribute to a better understanding of emotional functioning in this population (12). From a theoretical perspective, self-esteem may be linked to suppression of negative emotional expression through mechanisms related to social-evaluative threat, self-protection, and perceived interpersonal safety. Individuals with lower self-esteem may be more sensitive to rejection, criticism, discrimination, or exclusion and may therefore be more likely to inhibit the outward expression of anger, anxiety, or depressed mood in order to avoid further negative social evaluation. Among medical students, this tendency may be reinforced by academic pressure, professional socialization, contact with suffering, and expectations of emotional restraint. Therefore, the present study included a secondary exploratory indirect-association analysis to examine whether the relationship between self-esteem and individual dimensions of negative emotion suppression was statistically linked through other CECS dimensions. This analysis was hypothesis-generating and was not intended to establish causal or temporal mechanisms. The aim of the study was to assess the association between self-esteem and suppression of negative emotions among medical students in Poland.

The study also examined whether selected self-reported psychosocial characteristics were associated with differences in self-esteem and CECS scores. The following research questions were formulated:

1. What are the levels of self-esteem and suppression of negative emotions among medical students?
2. What is the association between self-esteem and suppression of negative emotions among medical students?
3. How do selected self-reported psychosocial characteristics differentiate SES and CECS scores?

## 2 Materials and methods

The sample consisted of 611 people aged 18–26 years. The mean age was 20.87 ± 1.72 years. All respondents were students of universities in Poland, representing medicine (26%) and health sciences (74%). The majority of respondents were women (77%). Most respondents came from large cities with more than 100,000 inhabitants (48.4%), followed by rural areas (21.4%), medium-sized cities with 20,000 to 100,000 inhabitants (15.4%), and small towns with fewer than 20,000 inhabitants (14.8%).

The study was conducted between March and May 2023 using a diagnostic survey method with questionnaires made available to students online. Data were collected using the CAWI procedure (Computer Assisted Web Interview). The study protocol was submitted to the Bioethics Committee of the Pomeranian Medical University in Szczecin, Poland. On the basis of the submitted documentation, the Committee stated that the study did not require formal ethics committee approval or opinion due to its anonymous questionnaire-based design (letter No. KB.006.123.2022/Z-10313, dated 14 November 2022). Participation was voluntary and anonymous, and informed consent was obtained from all participants before completing the online questionnaire.

The study used standardized tools such as the Rosenberg Self-Esteem Scale (SES) and the Emotion Control Scale (CECS), supplemented with an original survey questionnaire containing sociodemographic questions.

The Rosenberg Self-Esteem Scale (SES) measures an individual’s overall self-esteem. It is a two-dimensional scale: positive self-esteem (self-confidence or personal satisfaction) and negative self-esteem (self-contempt or personal devaluation). Scores in the range of 10–27 points indicate low self-esteem, 28–30 points indicate average self-esteem, and 32–40 points indicate high self-esteem (13). For the 10 SES questions regarding respondents’ self-esteem, Cronbach’s alpha coefficient in the study was 0.88.

The Courtauld Emotional Control Scale (CECS), originally developed by Watson and Greer and adapted into Polish by Juczyński, was used to assess the suppression of negative emotions (14, 15). The scale consists of three subscales assessing the tendency to control or inhibit the expression of anger, anxiety, and depressed mood (15). Higher CECS scores indicate greater suppression of negative emotional expression. In the present study, higher CECS scores were not interpreted as better or more adaptive emotion regulation, but as a stronger tendency to suppress the expression of negative emotions in difficult situations. For responses to the CECS survey, Cronbach’s alpha was 0.90. Dividing the CECS into CECS-anger, CECS-depression, and CECS-anxiety questions, Cronbach’s alpha was 0.85, 0.83, and 0.87, respectively. These values indicated good internal consistency of the scales used in the study.

Statistical analyses were performed using a significance level of *p* < 0.05. The distribution of SES and CECS scores was assessed using the one-sample Kolmogorov–Smirnov test. Since the distributions of SES scores (*p* = 0.065, z = 0.053) and CECS scores (*p* = 0.084, *z* = 0.051) did not significantly deviate from normality, parametric statistical tests were used. Differences between two groups were assessed using Student’s *t*-test, while differences according to place of residence were analyzed using one-way ANOVA. Pearson’s correlation coefficient was used to assess the relationship between SES and CECS scores. Regression analysis and the Sobel test were used to conduct secondary exploratory indirect-association analyses. This approach was based on general methodological recommendations for statistical mediation and indirect-effect analysis (16, 17). These analyses examined whether the association between self-esteem and each CECS subscale was statistically linked through the remaining CECS dimensions. Separate regression-based models were tested for CECS-anger, CECS-depression, and CECS-anxiety as dependent variables. The interpretation of indirect associations was guided by recommendations concerning the assessment and comparison of indirect effects in indirect-effect models (18).

The analyses were exploratory and hypothesis-generating. Because the study had a cross-sectional design and all variables were measured at the same time, the indirect associations were not interpreted as evidence of causal mediation, temporal ordering, or psychological mechanisms. This cautious interpretation is consistent with methodological recommendations emphasizing that statistically significant indirect effects do not by themselves establish a true mediator or causal mechanism (19).

The study was designed and reported as a cross-sectional observational study, with reference to the STROBE recommendations for reporting observational studies (20).

The original questionnaire included sociodemographic questions and three additional self-report items concerning selected psychosocial experiences. Self-reported traumatic experience was assessed using a single question: “Have you experienced any trauma in your life?” The examples provided included physical violence, psychological violence, stigmatization, loss of a loved one, and other subjectively significant experiences.

Perceived discrimination or social exclusion was assessed using a single question asking whether the respondent had experienced discrimination or social exclusion. Sense of belonging to a social group was assessed using the question: “Do you feel that you belong to any organized social group, such as a hobby group, student scientific club, or similar group?” These variables were interpreted as self-reported psychosocial experiences. The questions did not assess the type, severity, timing, duration, or clinical consequences of these experiences. These variables were not treated as clinical constructs or primary explanatory variables, but as broad self-reported contextual characteristics.

## 3 Results

Analyzing the responses received from 611 surveyed people, the average response value for the SES questionnaire is 28.22, and the standard deviation is 5.42, the response ranges in this questionnaire are 10–40. The average response score for the CECS questionnaire is 53.59 with a standard deviation of 12.49, the minimum point value is 25 and the maximum is 83. For both distributions, the skewness is negative and close to zero, so the distributions are close to a symmetrical distribution, for the SES distribution there is a slight advantage of higher values from the average. Detailed results of the SES and CECS surveys are presented in Table 1.

**Table 1:** Results of the SES and CECS questionnaires.

Questionnaire	*N*	Mean ± SD	Median ± QD	Min – Max	Skewness	Cronbach’s alpha
SES	611	28.22 ± 5.42	28 ± 4	10–40	− 0.17	0.88
CECS	611	53.59 ± 12.49	53 ± 9.5	25–83	− 0.01	0.90

*N*, number of people; SD, standard deviation.

The mean SES score was within the average range of self-esteem. The mean CECS score indicated a moderate tendency to suppress negative emotions in the study group. The results of the CECS questionnaire and its subscales (anger, depression and anxiety) were also analyzed due to the respondents’ subjective assessment of the need to suppress their emotions. Statistical tests showed a statistically significant difference in responses for the CECS questionnaire and its subscales for people who confirmed the need to suppress their emotions and those who denied such a need. A statistically significantly higher average value of the CECS indices (total, anger, depression, anxiety) was obtained for respondents indicating the need to suppress their emotions. For example, in the CECS-total results, the average value of the indicator is 58.28, while for people who do not feel the need to suppress emotions, the average value of the CECS-total indicator is 47.66 (Table 2). These results indicate consistency between respondents’ subjective declaration of the need to suppress emotions and higher CECS scores.

**Table 2:** CECS scores according to respondents’ subjective need to suppress emotions and perceived adequacy of emotional expression.

Question: Do you feel the need to suppress your emotions?						
Questionnaire	Answer	*N*	Mean ± SD	Min – Max	Stat	*p*
CECS—total	Yes	341	58.28 ± 11.10	31–83	11.5007	<0.001
	No	270	47.66 ± 11.61	25–76		
CECS—anger	Yes	341	19.1 ± 5.02	7–28	9.8014	<0.001
	No	270	15.19 ± 4.73	7–27		
CECS—depression	Yes	341	20.28 ± 4.28	8–28	10.0187*	<0.001
	No	270	16.29 ± 4.59	7–28		
CECS—anxiety	Yes	341	18.91 ± 5.34	7–28	6.1878	<0.001
	No	270	16.19 ± 5.45	7–28		

Question: Do you think that expressing your emotions is appropriate to the situation?						
Questionnaire	Answer	*N*	Mean ± SD	Min – Max	Stat	*p*
CECS—total	Yes	382	51.42 ± 11.66	25–80	−5.684	<0.001
	No	229	57.21 ± 13.01	25–83		
CECS—anger	Yes	382	16.79 ± 4.92	7–28	−3.5569	<0.001
	No	229	18.34 ± 5.65	7–28		
CECS—depression	Yes	382	17.72 ± 4.65	7–28	−5.35	<0.001
	No	229	19.84 ± 4.88	7–28		
CECS—anxiety	Yes	382	16.91 ± 5.39	7–28	−4.644	<0.001
	No	229	19.03 ± 5.58	7–28		

*N*, number of people; SD, standard deviation, * Mann–Whitney *U* test.

Responses to the CECS questionnaire and its subscales regarding anger, depression and anxiety were analyzed due to the answer to the question regarding the adequacy of expressing emotions in various situations. The *t*-test showed that, in all four cases (CECS-total, CECS-anger, CECS-depression, CECS-anxiety), CECS scores differed significantly according to respondents’ subjective assessment of the adequacy of their emotional expression. For people who declare that they do not express their emotions adequately to the situation, the average value of the indicator for the CECS questionnaire and its subscales is statistically significantly higher than the average value of the indicator of people declaring that they express their emotions adequately to the situation (Table 2).

Differences in SES and CECS scores were analyzed according to gender, place of residence, self-reported traumatic experiences, perceived discrimination or social exclusion, and sense of belonging to a social group. Detailed results are presented in Tables 3, 4.

**Table 3:** SES and CECS scores according to gender and place of residence.

SES	*N*	Mean ± SD	Min – Max	tstat	*p*
Gender					
Women	472	27.83 ± 5.32	10–40	−3.26	<0.05
Men	139	29.53 ± 5.56	14–40		
Domicile					
Big city	296	27.91 ± 5.49	10–40	1.1332	0.3349
Average city	94	28.85 ± 4.89	19–39		
A small town	90	28.8 ± 5.82	18–40		
Village	131	28.06 ± 5.31	15–40		

CECS	*N*	Mean ± SD	Min – Max	tstat	*p*
Gender					
Women	472	52.98 ± 12.52	25–83	−2.219	<0.05
Men	139	55.65 ± 12.21	25–80		
Domicile					
Big city	296	53.5 ± 12.07	25–83	1.9149	0.1259
Average city	94	51.49 ± 11.12	31–79		
A small town	90	53.32 ± 13.86	25–82		
Village	131	55.48 ± 13.21	28–83		

*N*, number of people; SD, standard deviation.

**Table 4:** SES and CECS scores according to selected self-reported psychosocial characteristics.

SES	*N*	Mean ± SD	Min – Max	tstat	*p*
Experiencing trauma					
Yes	367	27.22 ± 5.34	10–40	−5.739	<0.001
No	244	29.72 ± 5.18	16–40		
Experience of discrimination/social exclusion					
Yes	345	27.3 ± 5.53	10–39	−4.833	<0.001
No	266	29.4 ± 5.04	16–40		
A sense of belonging to a group					
Yes	301	29.39 ± 5.32	15–40	5.4172	<0.001
No	310	27.07 ± 5.27	10–39		

CECS	*N*	Mean ± SD	Min – Max	tstat	*p*
Experiencing trauma					
Yes	367	54.67 ± 13	25–83	2.6474	<0.05
No	244	51.95 ± 11.5	25–77		
Experience of discrimination/social exclusion					
Yes	345	54.51 ± 12.79	25–83	2.0784	<0.05
No	266	52.39 ± 12.02	25–83		
A sense of belonging to a group					
Yes	301	52.26 ± 12.7	25–82	−2.603	<0.05
No	310	54.88 ± 12.17	25–83		

*N*, number of people; SD, standard deviation.

Analysis of the answers using the *t*-test showed that there are statistically significant differences between the answers of women and men for both questionnaires used. Men scored higher than women in self-esteem and in suppression of negative emotions measured by CECS. The average value of the level of self-esteem in men according to SES was 29.53, while in women it was 27.83 (Table 3). The average value from the CECS survey on emotion control was 55.65 for men and 52.98 for women (Table 3).

It was also checked whether the results obtained from the questionnaires depended on the respondents’ place of residence. The one-way ANOVA statistical tests performed showed that the place of residence does not influence the respondents’ answers to the SES and CECS survey questions (Table 3).

SES and CECS scores differed according to selected self-reported psychosocial characteristics. Students declaring selected adverse psychosocial experiences had lower SES scores and higher CECS scores, whereas students reporting a sense of belonging to a social group had higher SES scores and lower CECS scores. These variables were based on single self-report items and should therefore be interpreted descriptively. Detailed results are presented in Table 4.

Using the Pearson correlation coefficient, the linear correlation of the results of the SES questionnaire with the results of the CECS-total, CECS-anger, CECS-depression and CECS-anxiety questionnaires was analyzed. Statistically significant, moderate negative linear correlation was obtained for the results of the SES and CECS-depression questionnaires (*r* = − 0.4, *p* < 0.001) and SES and CECS-total survey (*r* = − 0.37, *p* < 0.001). A statistically significant, weak negative correlation was obtained for the results of the SES and CECS-anger (*r* = − 0.26, *p* < 0.001). Additionally, the tests showed a statistically significant, weak negative correlation between the results of the SES questionnaires and CECS-anxiety (*r* = − 0.24, *p* < 0.001). Detailed results of Pearson’s linear correlation are presented in Table 5.

**Table 5:** Pearson correlations between SES and CECS scores.

Questionnaire	CECS—total	CECS—anger	CECS—depression	CECS—anxiety
SES	*r* = − 0.37	*r* = − 0.26	*r* = − 0.4	*r* = − 0.24
	*p* < 0.001	*p* < 0.001	*p* < 0.001	*p* < 0.001
	*n* = 611	*n* = 611	*n* = 611	*n* = 611

Overall, the results showed a consistent pattern in which lower self-esteem was associated with higher suppression of negative emotions. Students reporting selected adverse psychosocial experiences had lower SES scores and higher CECS scores. Conversely, students declaring a sense of belonging to a social group had higher self-esteem and lower suppression of negative emotions. These findings indicate an associative pattern linking self-esteem, adverse psychosocial experiences, group belonging, and suppression of negative emotions among medical students. Secondary exploratory indirect-association analyses were conducted to examine whether the relationship between SES and individual CECS subscales was statistically linked through the remaining CECS dimensions. Across the tested models, lower SES was associated with higher suppression of anger, anxiety, and depressed mood, and the CECS subscales were strongly interrelated. The strongest indirect statistical associations involved CECS-depression, particularly in the models with CECS-anger and CECS-anxiety as dependent variables. Because all CECS subscales belong to the same instrument and were measured at the same time, these findings should be interpreted as exploratory within-scale indirect associations rather than as evidence of causal mediation. Detailed regression coefficients and Sobel test results are provided in Supplementary Tables S1–S3.

## 4 Discussion

The present study examined the association between self-esteem and suppression of negative emotions among medical students. The main finding was that lower self-esteem was associated with higher CECS scores, both in the total score and in the anger, depression, and anxiety subscales. In addition, students who reported selected adverse psychosocial experiences had lower self-esteem and higher suppression of negative emotions, whereas students reporting a sense of belonging to a social group had higher self-esteem and lower CECS scores. However, these findings should be interpreted cautiously because these characteristics were assessed using broad single-item self-report questions. These findings should be interpreted as associations and not as evidence of causal relationships. The secondary exploratory indirect-association analyses should be understood within a cautious theoretical framework. Lower self-esteem may be associated with greater sensitivity to social-evaluative threat and with a stronger tendency to protect the self by inhibiting the expression of negative emotions. In medical students, this tendency may be reinforced by academic pressure, professional norms of emotional restraint, and the need to function in emotionally demanding educational and clinical contexts. The observed indirect associations among CECS-anger, CECS-depression, and CECS-anxiety may therefore reflect a broader pattern of co-occurring suppression of negative emotional expression rather than distinct causal pathways between separate psychological constructs. Accordingly, these findings should be treated as hypothesis-generating and require confirmation in longitudinal studies using temporally ordered measures.

These secondary exploratory analyses generate several hypotheses for future research rather than conclusions about causal mechanisms. First, longitudinal studies should test whether lower self-esteem predicts an increase in suppression of negative emotional expression over time among medical students. Second, future studies should examine whether suppression of depressed mood functions as a central component of a broader pattern of emotional inhibition linking self-esteem with the suppression of anger and anxiety. Third, future research should assess whether perceived discrimination, social exclusion, and lack of group belonging intensify the association between lower self-esteem and suppression of negative emotions. These hypotheses require longitudinal or prospective designs and should be tested using multidimensional measures of psychosocial adversity, social support, and emotion-regulation strategies.

The present findings may also be interpreted in the broader context of polycrisis. Contemporary medical students enter higher education during a period marked by overlapping health, social, economic, geopolitical, and environmental stressors, including the long-term consequences of the COVID-19 pandemic, the war in Ukraine, economic uncertainty, and growing concerns about mental health among young adults (21, 22). A polycrisis perspective emphasizes that such stressors should not be viewed in isolation, because their cumulative and interacting effects may increase psychological vulnerability (21). In this context, self-esteem and the tendency to suppress negative emotions may be important psychosocial indicators of how young adults adapt to complex and prolonged uncertainty (22). However, because the present study did not directly measure exposure to specific crises, these interpretations should be treated as contextual rather than causal.

Self-esteem is defined as a subjective personal evaluation of oneself that influences interactions and feelings towards oneself and others. It is largely related to social support (23). The importance of self-esteem is emphasized by decades of theory development and research confirming its relationship, e.g., with mental wellbeing and life satisfaction (24). High self-esteem is also associated with self-respect and self-acceptance. It is a protective factor against unhealthy behaviours such as anxiety, depression and even suicidal thoughts (25). According to the study by Calero et al. (26), having high self-esteem and self-concept in adolescence is significantly related to being emotionally stable and a social and responsible entity. It is assumed that when faced with difficult life circumstances, challenges and adversities, people with higher self-esteem show more positive coping and adjustment strategies, which may further protect the individual’s health and wellbeing (27, 28).

In turn, people with low self-esteem may be perceived as having less self-confidence and less ability to identify resources for intended goals (29). Therefore, self-esteem may be considered an important psychosocial correlate of emotional functioning in young adults, although the present study does not allow conclusions about the effectiveness of preventive or therapeutic interventions. The average result of the Rosenberg Self-Esteem Scale in the group of surveyed students was 28.22 ± 5.42 points, which is the lower limit of the average level of self-esteem and is lower than the result obtained in similar studies almost 20 years ago (30). Over the last 20 years, many studies have examined gender-, age-, lifespan-, and birth-cohort-related differences in self-esteem (31–33, 59, 60).

The conclusion that emerges from the research conducted so far is a significant difference between the sexes, and precisely men tend to report a higher level of self-esteem than women. This gender difference emerges during adolescence and persists throughout early and middle adulthood before decreasing and perhaps even disappearing in old age. Reported effect sizes typically fall within the small to medium effect range. In a meta-analysis of 216 effect sizes, Kling et al. (31) found an overall effect size of d = 0.21 across all age groups, with the largest effect occurring in late adolescence (*d* = 0.33). The surveyed men were characterized by a significantly higher level of self-esteem than women, which is consistent with previous research conducted among young people in various countries around the world (24, 29, 34, 35). Despite clear reports on gender differences in the level of self-esteem of young adults, regardless of the living environment and culture, the reasons why men perceive themselves better in terms of self-esteem have not been clearly explained.

Life experiences, including adverse or subjectively traumatic experiences, may also be associated with self-esteem. Cumulative and severe exposure to adversity may lead to the development of poor, low and negative self-esteem, as well as social withdrawal (36). Childhood trauma, defined as emotional, physical and sexual abuse or emotional and physical neglect before the age of 16, has been extensively researched and found to be a significant cause of the development and deterioration of mental health in adulthood (37, 38). Previous research confirms the relationship between the impact of traumatic experiences, related to, among others, stigmatization of an individual, on the process of shaping and the level of self-esteem (39, 40) and mental health (41). There are also reports indicating that the negative impact of trauma on the mental health of children and adolescents resulted in a lower level of self-esteem (42). Additionally, experiences of discrimination in adolescence are associated with lower self-esteem in adulthood (43), suggesting that experiences of stigma early in life may have lasting effects on self-esteem.

In our study, students who declared experience of a traumatic situation obtained significantly lower SES scores compared to people without such experiences, which is consistent with the results of other authors (44). However, the present study did not assess trauma using a validated trauma checklist or clinical diagnostic measure. Therefore, the findings should be interpreted in relation to self-reported traumatic experiences rather than clinically verified trauma exposure or trauma-related psychopathology. The observed differences indicate that students who perceived themselves as having experienced trauma showed lower self-esteem and higher suppression of negative emotions, but the study does not allow conclusions about the type, severity, timing, or psychological consequences of these experiences.

As social beings, humans have a basic “need to belong,” and the desire for meaningful relationships is important for perceptions of quality of life throughout the lifespan (45). Social acceptance and belonging to a peer group are particularly important during adolescence (46). In psychology, it has long been assumed that social relationships play a key role in shaping an individual’s self-esteem (47). Attachment to a group and external attribution of blame may protect self-esteem to some extent. People seeking social support and using proactive strategies to cope with stress are characterized by higher self-esteem (48, 49). The present findings are consistent with this perspective, as students who reported belonging to a social group had higher SES scores and lower CECS scores. However, due to the cross-sectional design, it cannot be determined whether group belonging supports self-esteem or whether students with higher self-esteem are more likely to engage in social groups. Young adults scored significantly higher in the SES questionnaire if they declared that they belonged to any social group.

Emotions are considered one of the key mechanisms of human functioning and adaptation to the requirements of the environment. Well-researched emotion regulation strategies include reappraisal and suppression. Despite its widespread use, emotion suppression is associated with psychological, social, cognitive and physiological costs (50). In this context, higher CECS scores should not be interpreted as better emotional competence or more effective emotion regulation. They indicate a stronger tendency to inhibit the expression of anger, anxiety, and depressed mood. It has been found that the lack of proper regulation of emotions is strongly associated with the occurrence of mental health problems (51). Empirical evidence has supported the mediating role of cognitive emotion regulation strategies in the relationship between traumatic experiences and mental health problems in various populations, including a clinical sample (52), a student sample (53), and children and adolescents (54, 55). Previous studies suggest that traumatic experiences may be associated with later difficulties in emotion regulation (55, 56).

The assessment of emotion control in terms of suppressing negative emotions used in our study showed that a large number of young adults subjectively feel the need to suppress their own emotions and the inadequacy of expressing them. These people also obtained a CECS scale result indicating a greater degree of suppression of negative emotions than the other respondents. It has also been shown that men suppress their anger, anxiety and depression more often than women. Moreover, selected self-reported psychosocial characteristics were associated with higher CECS scores among the surveyed students. Young adults with higher self-esteem showed lower suppression of anger, depression, and anxiety. This pattern suggests that self-esteem may be associated with a lower tendency to inhibit the expression of negative emotions, although the cross-sectional design does not allow conclusions about the direction of this relationship (57, 58). The mean SES score in the study group was located at the lower end of the average range, which may indicate the need for further research on self-esteem among medical students. A high proportion of respondents reported experiences they perceived as traumatic; however, this result should be interpreted cautiously because trauma was assessed using a single self-report item.

## 5 Limitations

Several limitations should be noted. First, the study had a cross-sectional design; therefore, the observed relationships should be interpreted as associations and not as evidence of causal or temporal effects. It is not possible to determine whether lower self-esteem contributes to greater suppression of negative emotions or whether a stronger tendency to suppress emotions is related to lower self-esteem over time. Second, the study was based on self-report measures, which may be affected by recall bias, social desirability, response errors, and individual differences in naming emotions and interpreting personal experiences.

Third, another limitation concerns the assessment of selected psychosocial characteristics. Self-reported traumatic experience, perceived discrimination or social exclusion, and sense of belonging to a social group were assessed using single broad self-report items. Therefore, these variables should be interpreted only as general subjective contextual characteristics and not as clinical or multidimensional measures. In particular, the trauma item did not assess the type, severity, timing, duration, recurrence, or clinical consequences of traumatic experiences. Fourth, the CECS measures the suppression or control of the expression of anger, anxiety, and depressed mood. It does not assess the full range of emotion-regulation strategies, such as cognitive reappraisal, acceptance, avoidance, or problem-focused coping.

Therefore, conclusions should be limited to suppression of negative emotions and should not be generalized to emotion regulation as a whole. Fifth, although secondary exploratory indirect-association analyses were conducted, the cross-sectional design prevents causal or temporal interpretation of the observed indirect associations. Moreover, because CECS-anger, CECS-depression, and CECS-anxiety are closely related subscales of the same instrument, these analyses should be understood as exploratory within-scale statistical models rather than as evidence of causal psychological mechanisms or independent psychological pathways. Finally, the sample consisted of students of medical and health sciences universities in Poland, with a predominance of women. This limits the generalizability of the results to other populations, age groups, educational contexts, and cultural settings. Therefore, the results and conclusions should be interpreted in relation to the surveyed group and may provide a basis for future studies conducted in larger and more diverse samples.

## 6 Conclusion

Lower self-esteem was associated with greater suppression of negative emotions among medical students. This association was observed for the CECS total score and for the suppression of anger, anxiety, and depressed mood. Selected self-reported psychosocial characteristics were also associated with differences in SES and CECS scores, but these findings should be interpreted descriptively and with caution. Due to the cross-sectional design, the results should not be interpreted causally. Future longitudinal studies using validated multidimensional measures are needed to verify the direction and stability of these associations.

## Data Availability

The raw data supporting the conclusions of this article will be made available by the authors, without undue reservation.
