# Supplementary material for: Self-esteem and suppression of negative emotions among medical students: a cross-sectional study
Source: Front Public Health. 2026 Aug 4;14:1846894. doi: 10.3389/fpubh.2026.1846894 (PMC13481214; doi:10.3389/fpubh.2026.1846894)
Supplement: Supplementary file 1 [file Supplementary_File_1.docx]

Supplementary Materials

Detailed results of secondary exploratory indirect-association analyses. Secondary exploratory indirect-association analyses were conducted to examine whether the associations between self-esteem and individual CECS subscales were statistically linked through the remaining CECS dimensions. In the first set of models, SES was treated as the independent variable and CECS-anger as the dependent variable, while CECS-depression and CECS-anxiety were included separately in the indirect-association models.

The results were consistent with the correlation analysis. Higher SES scores were associated with lower CECS-depression scores (b = -0.36, p < 0.001) and lower CECS-anxiety scores
(b = -0.25, p < 0.001). CECS-depression and CECS-anxiety were positively associated with CECS-anger (b = 0.63, p < 0.001 and b = 0.30, p < 0.001, respectively). When CECS-depression was included in the model, the direct association between SES and CECS-anger was no longer statistically significant (b = -0.036, p = 0.306), while CECS-depression remained significantly associated with CECS-anger (b = 0.61, p < 0.001). The Sobel test indicated a statistically significant indirect association through CECS-depression (Z = -9.173, p < 0.001). When CECS-anxiety was included in the model, the association between SES and CECS-anger remained statistically significant but was reduced (b = -0.19, p < 0.001), and the Sobel test also indicated a statistically significant indirect association through CECS-anxiety (Z = -4.92, p < 0.001). Detailed results are presented in Supplementary Table S1.

**Supplementary Table S1.** Regression results for the secondary exploratory indirect-association analysis with SES as the independent variable and CECS-anger as the dependent variable.

| Mediator | Association between SES and CECS-anger [b, SE] | Association between SES and mediator [b, SE] | Association between mediator and CECS-anger [b, SE] | Indirect association of SES with CECS-anger (A) with mediator (B) [b, SE] | Sobel test [Z-stat, SE] |
| --- | --- | --- | --- | --- | --- |
| CECS - depression | - 0.26, 0.038 (p< 0.001) | - 0.36, 0.033 (p<0.001) | 0.63, 0.036 (p<0.001) | (A) - 0.036, 0.035 (p=0.306)  (B) 0.61, 0.039 (p<0.001) | -9.173, 0.0245 (p<0.001) |
| CECS - anxiety | - 0.26, 0.038 (p< 0.001) | - 0.25, 0.04 (p<0.001) | 0.3, 0.036 (p<0.001) | (A) - 0.19, 0.038 (p<0.001)  (B) 0.26, 0.037 (p<0.001) | -4.92, 0.015 (p<0.001) |

Note: b — unstandardized regression coefficient; SE — standard error. These analyses were exploratory and should be interpreted as indirect statistical associations rather than as evidence of causal mediation, temporal ordering, or psychological mechanisms.
In the second set of models, SES was treated as the independent variable and CECS-depression as the dependent variable, while CECS-anger and CECS-anxiety were included separately
in the indirect-association models. SES was negatively associated with CECS-depression
(b = -0.36, p < 0.001). CECS-anger and CECS-anxiety were positively associated with CECS-depression. When CECS-anger was included in the model, the direct association between SES and CECS-depression was reduced but remained statistically significant (b = -0.24, p < 0.001), while CECS-anger was significantly associated with CECS-depression (b = 0.47, p < 0.001).
The Sobel test indicated a statistically significant indirect association through CECS-anger
(Z = -6.277, p < 0.001). When CECS-anxiety was included in the model, the direct association between SES and CECS-depression also remained statistically significant (b = -0.27, p < 0.001), and the Sobel test indicated a statistically significant indirect association through CECS-anxiety (Z = -5.55, p < 0.001). Detailed results are presented in Supplementary Table S2.

**Supplementary Table S2.** Regression results for the secondary exploratory indirect-association analysis with SES as the independent variable and CECS-depression as the dependent variable.

| Mediator | Association between SES and CECS- depression  [b, SE] | Association between SES and mediator [b, SE] | Association between mediator and CECS- depression [b, SE] | Indirect association of SES with CECS- depression (A) with mediator (B) [b, SE] | Sobel test  [Z-stat, SE] |
| --- | --- | --- | --- | --- | --- |
| CECS - anger | - 0.36, 0.033 (p<0.001) | - 0.26, 0.038 (p< 0.001) | 0.53, 0.03 (p<0.001) | (A) - 0.24, 0.029 (p<0.001)  (B) 0.47, 0.03 (p<0.001) | -6.277, 0.022 (p<0.001) |
| CECS - anxiety | - 0.36, 0.033 (p<0.001) | - 0.25, 0.04 (p<0.001) | 0.42, 0.031 (p<0.001) | (A) - 0.27, 0.031 (p<0.001)  (B) 0.35, 0.03 (p<0.001) | -5.55, 0.018 (p<0.001) |

Note: b — unstandardized regression coefficient; SE — standard error. These analyses were exploratory and should
be interpreted as indirect statistical associations rather than as evidence of causal mediation, temporal ordering, or psychological mechanisms.

In the third set of models, SES was treated as the independent variable and CECS-anxiety as the dependent variable, while CECS-anger and CECS-depression were included separately in the indirect-association models. SES was negatively associated with CECS-anxiety (b = -0.25, p < 0.001). CECS-anger and CECS-depression were positively associated with CECS-anxiety (b = 0.34, p < 0.001 and b = 0.55, p < 0.001, respectively). When CECS-anger was included in the model, the direct association between SES and CECS-anxiety was reduced but remained statistically significant (b = -0.17, p < 0.001), and the Sobel test indicated
a statistically significant indirect association through CECS-anger (Z = -5.24, p < 0.001).
When CECS-depression was included in the model, the direct association between SES
and CECS-anxiety was no longer statistically significant (b = -0.06, p = 0.139), while CECS-depression remained significantly associated with CECS-anxiety (b = 0.53, p < 0.001).
The Sobel test indicated a statistically significant indirect association through CECS-depression (Z = -8.405, p < 0.001). Detailed results are presented in Supplementary Table S3.

**Supplementary Table S3.** Regression results for the secondary exploratory indirect-association analysis with SES as the independent variable and CECS-anxiety as the dependent variable.

| Mediator | Association between SES and CECS- anxiety [b, SE] | Association between SES and mediator [b, SE] | Association between mediator and CECS- anxiety [b, SE] | Indirect association of SES with CECS- anxiety (A) with mediator (B) [b, SE] | Sobel test [Z-stat, SE] |
| --- | --- | --- | --- | --- | --- |
| CECS - anger | - 0.25, 0.04 (p<0.001) | - 0.25, 0.038 (p<0.001) | 0.34, 0.04 (p<0.001) | (A) - 0.17, 0.04 (p<0.001)  (B) 0.29, 0.041 (p<0.001) | -5.24, 0.017 (p<0.001) |
| CECS - depression | - 0.25, 0.04 (p<0.001) | - 0.36, 0.033 (p<0.001) | 0.55, 0.041 (p<0.001) | (A) -0.06, 0.04 (p=0.139)  (B) 0.53, 0.044 (p<0.001) | -8.405, 0.023 (p<0.001) |

Note: b — unstandardized regression coefficient; SE — standard error. These analyses were exploratory and should
be interpreted as indirect statistical associations rather than as evidence of causal mediation, temporal ordering, or psychological mechanisms.
